# The effectiveness of Collaborative Online International Learning (COIL) on intercultural competence development in higher education

**DOI:** 10.1186/s41239-022-00373-3

**Published:** 2023-01-24

**Authors:** Simone Hackett, Jeroen Janssen, Pamela Beach, Melanie Perreault, Jos Beelen, Jan van Tartwijk

**Affiliations:** 1 Department of Education and Pedagogy, Faculty of Social and Behavioural Sciences, Utrecht University, Heidelberglaan 1, 3584 CS Utrecht, The Netherlands; 2 Faculty of Health, Nutrition and Sports, The Hague University of Applied Sciences (THUAS), Johanna Westerdijkplein 75, 2521 EN The Hague, The Netherlands; 3 Department of Kinesiology, Sports Studies and Physical Education, The College at Brockport, State University of New York, New York, USA; 4 Global Learning Lectorate, The Hague University of Applied Sciences, The Hague, The Netherlands

**Keywords:** Collaborative Online International Learning (COIL), Collaborative learning, Intercultural competence, Internationalisation, Cultural intelligence

## Abstract

In this study we measured the effect of COIL on intercultural competence development using a quasi-experimental design. Our sample consisted of 108 undergraduate students from two universities, one located in the Netherlands (NL) and one in the United States (US). Students’ self-reported intercultural competence was measured using a pre-post survey which included the Cultural Intelligence Scale (CQS) and Multicultural Personality Questionnaire (MPQ). Qualitative data were collected to complement our quantitative findings and to give a deeper insight into the student experience. The data showed a significantly bigger increase in intercultural competence for the US experimental group compared to the US control group, supporting our hypothesis that COIL develops intercultural competence. This difference was not observed for the NL students, possibly due to the NL control group being exposed to other international input during the course.

**Supplementary Information:**

The online version contains supplementary material available at 10.1186/s41239-022-00373-3.

## Highlights

- We investigated the effectiveness of Collaborative Online International Learning (COIL).
- This is one of the first studies to use a quasi-experimental design using a control group to test the effectiveness of COIL.
- Both quantitative (survey) and qualitative data (reflection reports, focus group interviews) were used to examine the effectiveness of COIL.
- COIL significantly increases intercultural competence in terms of cultural intelligence.
- This increase was not observed for students who were already exposed to international experiences.

**Supplementary Information:**

The online version contains supplementary material available at 10.1186/s41239-022-00373-3.

## Introduction

Collaborative Online International Learning (COIL), an educational approach using online technology, has become a popular tool within universities around the world to help internationalise the curriculum (Rubin, 2017). COIL can facilitate students’ intercultural competence development at their home institute and therefore could be a way of ensuring all students have the opportunity to develop intercultural competencies, not just the select few who avail of a study or internship abroad. COIL falls under the social-constructivist educational approach of collaborative learning and places a focus on learning through social interaction, which is a keystone to developing intercultural competence (Guth & Rubin, 2015). COIL is a relatively new method, and literature and research on COIL is still emerging (Kastler & Kyle, 2020). Consequently, there are few experimental studies investigating the effectiveness of COIL and to our knowledge no studies have been carried out that make use of a control group to assess the effect of COIL on intercultural competence development. Therefore, the purpose of this study is to empirically test the effectiveness of COIL on students’ intercultural competence development through a quasi-experimental design.

### Internationalisation of education

Internationalisation of higher education is defined as “the intentional process of integrating an international, intercultural or global dimension into the purpose, functions and delivery of post-secondary education, in order to enhance the quality of education and research for all students and staff, and to make a meaningful contribution to society” (de Wit & Hunter, 2015, p. 3). Internationalisation has become common practice at many Higher Education Institutions (HEIs) around the world (de Wit et al., 2015). HEIs internationalise for social, cultural, political, academic, and economic reasons (Knight, 2004), but one of the common rationales for internationalising is to ensure HEIs produce “global-ready” graduates who have acquired the intercultural competencies to be able to address issues associated with global developments and challenges (Deardorff & Jones, 2012; van Gaalen & Gielesen, 2014). These competencies include attributes such as respect, openness, curiosity, cultural knowledge, and skills such as the ability to observe, listen, evaluate, and interpret (Deardorff, 2006).

Most HEIs support intercultural competence development through student mobility, i.e., offering students the opportunity to study abroad at an international partner university or do an international internship abroad during their studies. Consequently, student mobility tends to dominate most HEIs internationalisation strategies (de Wit & Hunter, 2015). However, due to concerns over finance, language proficiency or for personal reasons, only a small minority of students go abroad during their academic career (Findlay et al., 2016; Mol & Timmerman, 2014; Rostovskaya et al., 2020). Both in Europe and the United States it is reported that on average only 10–13% of undergraduates have studied or worked abroad during their studies (European Commission, 2020; Institute for International Education (IIE), 2020; NAFSA, 2018; Teichler, 2019). Therefore, although this approach of travelling abroad facilitates intercultural competence development (Souto-Otero, 2019), it is not an inclusive educational practice, as most students do not avail of it.

In addition, during the COVID-19 pandemic many HEIs had to make a rapid shift to online teaching and learning which led HEIs to discover the potential of online educational practices but also explicitly showed the vulnerability of overreliance on student mobility to help students acquire intercultural competencies. There are also rising concerns over the carbon footprint and environmental impact of student and staff mobility (Shields, 2019). These developments have resulted in a surge of interest in COIL as an alternative way to internationalise the curriculum and in some cases even replace student mobility (Liu & Shirley, 2021).

### Collaborative Online International Learning (COIL)

The term “COIL” was coined by the State University New York in 2006 and the characteristics of COIL have been described by Rubin (2017), State University New York’s COIL Center (SUNY 2020) and Rubin and Guth (2022). COIL falls under the term Virtual Exchange (VE) which O’ Dowd (2018) describes as an umbrella term to cover various online teaching and learning approaches that focus on online intercultural interactions and collaboration. In this study we do not label the COIL practice simply as VE, as this term is too broad and is not explicit enough in describing the elements, the learning experience or outcomes of COIL.

There are several characteristics which make COIL unique compared to other VE initiatives. The term COIL is transparent and explicit in describing the practice and highlights the essence of the approach i.e., collaborative learning. COIL promotes collaborative learning for both educators and students (Rubin, 2017). This involves two or more educators, who are working at geographically separated institutions, connecting and collaborating (online) to design a shared syllabus for their students, including joint online group assignments with co-created learning outcomes. In addition, COIL focuses on subject knowledge as well as intercultural competence development and can be implemented within courses in a broad range of disciplines.

A COIL course, which can last anywhere between four weeks to a whole semester, usually involves the creation of multicultural teams, made up of students from both institutions who connect online to work on group assignments. During this process, educators intentionally design assignments with intercultural learning in mind and facilitate and promote this collaborative intercultural learning throughout the COIL course. The goal of this collaboration is to broaden students’ understanding of course content and to help them develop intercultural competencies. In doing so, COIL offers students an authentic international learning experience at their home institution rather than at an institution abroad.

### Collaborative Learning

COIL is based on the collaborative learning (CL) educational approach to teaching and learning which involves two or more students working together to solve a problem, complete a task, or create a product either in a face to face or online setting (Dillenbourg, 1999; Laal & Laal, 2012). During CL the focus is on learning and building on knowledge through social interaction. In this framework, the educator acts as a facilitator for students, who are working on a collaborative task, while the students serve as experts for one another and learn through interacting with each other, constructing and building on knowledge (Davidson & Major, 2014; O’Donnell & O’Kelly, 1994). CL is a well-researched educational approach and there is a consensus amongst most researchers that it can have a positive impact on student achievement (Slavin, 1990), facilitates the development of employability skills (Chan et al., 2014) and increases openness to diversity (Cabrera et al., 2002). Research into CL has also shown that students who have collaborated in group work, specifically in multicultural teams in the physical classroom, have developed intercultural competencies (De Hei et al., 2020; Liang & Schartner, 2020). There is also some evidence that this is also the case for students within the online classroom (Erez et al., 2013).

Research into COIL is a growing field but the literature available on COIL mostly consists of descriptions of best practices, case-studies, recommendations on implementation or evaluations (de Castro et al., 2018; Esche, 2018; Katre, 2020; Marcillo-Gómez & Desilus, 2016; Mundel, 2020; Rebek, 2022; West et al., 2022). While these case-studies and descriptions may provide practitioners with valuable information, they do not seek to empirically test the effectiveness of COIL on intercultural competence development, and their results cannot be generalized.

Empirical research studies on Virtual Exchange (VE) and specifically the COIL approach are scarce, and research that has been carried out tends to be exploratory, qualitative in nature or small scale. Baroni et al. (2019) applied an experimental design to measure the impact of VE on a sample of foreign language pre-service student teachers. Baroni et al.’s study, while employing a large sample and involving many classes and students collaborating, provided mixed results that could be due the fact that for their study it was difficult to control for important extraneous variables (e.g., impact of differences between courses and assignments). Nevertheless, other studies have provided us with interesting insights on student perspectives and experiences. An exploratory study found that students who followed a COIL course had a positive attitude toward international online collaboration but also found that diversity in communication styles may hinder successful learning experiences (Kayumova & Sadykova, 2016). Naicker et al. (2021) found that students are more open to learning about other cultures, different religions and traditions after a COIL experience. Vahed and Rodriguez (2020) discovered that COIL positively influenced intercultural awareness and stimulated students to be globally engaged. However, a limitation of these studies is the absence of a control group to compare findings. To our knowledge, there is no controlled study that has measured intercultural competence development specifically as a result of COIL.

### Assessing Intercultural Competence

There have been many definitions and models developed to describe and explain intercultural competence and various tools developed to assess it (see Spitzberg & Changnon, 2009 and Griffith et al., 2016 for an overview). In this study, intercultural competence is defined as “the ability to communicate effectively and appropriately in intercultural situations based on one’s intercultural knowledge, skills and attitudes” (Deardorff, 2006, p. 247).

Within this study, two models i.e., Cultural Intelligence (CQ) developed by Earley and Ang, (2003) and the Multicultural Personality model developed by van der Zee and van Oudenhoven (2000) have been selected to explore and assess the conditions to adjust and communicate effectively in intercultural situations. These models have been selected as both include validated assessment tools, i.e., Cultural Intelligence Scale (CQS) and Multicultural Personality Questionnaire (MPQ) and both have been shown to have the most promising evidence for assessing intercultural competence (Leung et al., 2014; Matsumoto & Hwang, 2013).

*Cultural Intelligence* (CQ): Defined as “an individual’s capability to function and manage effectively in culturally diverse settings” (Ang et al., 2007, p. 336), CQ is a concept which considers intercultural capabilities as a form of specific intelligence that can be measured and developed. CQ has been used widely to examine and assess intercultural competence across various educational contexts (Engle & Crowne, 2014; Erez et al., 2013; Lin et al., 2012; Varela & Gatlin-Watts, 2014). The CQ model incorporates four constructs which include: *Metacognitive*, which reflects an individual’s mental ability to acquire and comprehend cultural knowledge and use cognitive strategies in intercultural situations. *Cognitive*, which reflects an individual’s knowledge about cultures and cultural differences. *Motivational*, which reflects an individual’s drive and intrinsic interest in other cultures and intercultural situations. *Behavioural*, which reflects an individual’s ability to utilise skills to communicate and interact with flexibility in cross-cultural interactions.

*Multicultural Personality*: Research has shown that individuals’ personality traits vary from each other, and these differences influence individuals’ thoughts and behaviour especially when adapting and communicating effectively in intercultural situations (Brisset et al., 2010; Wang & Ratanasiripong, 2010; Ying & Han, 2006). Given this, much attention has been given to what traits or personal attributes are better able to adjust to intercultural situations (Leung et al., 2014). Van der Zee and van Oudenhoven’s Multicultural Personality model (2000) continues to be one of the most popular and robust models used to examine the relationship between personality traits and the ability to communicate effectively and appropriately in intercultural situations (Hofhuis et al., 2020a; Matsumoto & Hwang, 2013; Ponterotto, 2014). The model defines multicultural effectiveness as “success in the fields of professional effectiveness, personal adjustment and intercultural interactions” (Van der Zee & van Oudenhoven’s, 2000, p.293). Researchers have used the model in various contexts to measure intercultural competence development (Leong, 2007; Schartner, 2016; van der Poel, 2020) and to measure intercultural competence development as a result of culturally mixed group work (Liang & Schartner, 2020). The model’s assessment tool identifies five personal attributes related to effectiveness in intercultural situations: *Cultural Empathy:* refers to the ability to empathize with feelings, thoughts, and behaviours of members from different cultural groups. *Flexibility:* ability to switch easily from one behavioural strategy to another. *Social Initiative:* tendency to actively approach and interact in social situations, initiating conversation instead of taking a back seat. *Open-mindedness:* an open and unprejudiced attitude towards people from different cultures or different cultural norms and values. *Emotional Stability:* reflects ability to remain calm in stressful situations versus a tendency to show strong emotional reactions under stressful circumstances.

By using the CQS and MPQ models as a basis for this study, we hope to get deeper insight into the effectiveness of COIL on intercultural competence by examining a combination of capabilities (as assessed using the CQS) and personal attributes (assessed using the MPQ).

## Aim of the study

The purpose of this study is to empirically test, using a controlled quasi-experiment design, the effect of a COIL intervention on intercultural competence development. Quasi experimental designs are helpful in describing the effects of an intervention and play an important role within computer-supported collaborative learning (Janssen & Kollar, 2021). In the current study, the presence of control groups, helps us confirm that the results of our study are due to COIL rather than any extraneous variables. Such research provides us with the most reliable evidence of the effectiveness of COIL on students’ intercultural competence development. Without a control group we have no knowledge of whether an intervention such as COIL is effective or not. Similar concerns have been raised by Rienties et al (2020) who call for further empirical research into Virtual Exchange using control groups to measure any positive or negative findings. This call has been reiterated by more researchers in the field of COIL and Virtual Exchange (Chang & Shinnar, 2022; Zak, 2021). Therefore, this study responds to these calls.

In this study, we test the following hypothesis: students gain scores on intercultural competence from before a course to after the course will be bigger for students within the experimental groups (COIL groups) compared to students within the control groups. We hope that the results of this study will provide empirical evidence that COIL does indeed have the intended effect and helps students develop intercultural competence.

## Materials and methods

### Design

We used a quasi-controlled experiment design consisting of four groups: a US experimental group (*N* = 30), a NL experimental group (*N* = 24), a US control group (*N* = 31) and a NL control group (*N* = 24). Each group was taught by a different educator. Classes were assigned randomly to either the experimental or the control group. As described in the introduction, we used existing measurement tools i.e., Cultural Intelligence Scale (CQS) and Multicultural Personality Questionnaire (MPQ), which were given to all students before and after the course. Given its complexity, intercultural competence should be assessed using a mixed-method approach (Deardorff, 2011). Therefore, we also collected qualitative data through focus group interviews and written student reflection reports. In addition, combining both quantitative and qualitative methods has been argued to be advantageous as they cancel out each other’s weaknesses and result in superior research (Johnson & Onwuegbuzie, 2016).

### Participants

Before the study took place, we conducted a power analysis to identify the required sample size for this study and a sample size of 102 was required to obtain a power of 0.80 in detecting a medium effect size of 0.5 (at *p* = 0.05). This study involved a sample of 108 undergraduate students from two universities who took part in a project course. The sample included 48 students from the university in the Netherlands (NL) and 60 students from the university in the United States (US). Details on students’ gender and nationalities can be found in Table 1.

**Table 1:** Student gender and nationalities

Group	*n*	Males	Females	Nationality
US experimental	29	65%	35%	100% US
US control	31	66%	34%	100% US
NL experimental	24	19%	81%	71% Dutch 29% non-Dutch*
NL control	24	29%	71%	83% Dutch 17% non-Dutch**
Total	108			

*Students came from the US, Slovakia, Lithuania, Hungary, Poland, Germany, Italy

**Students came from the UK, Bulgaria, Slovakia and Brazil

Students from all groups were registered in undergraduate bachelor programmes at the NL and US university. For the NL groups, the project course in which the experiment took place was part of an elective minor. The minor was English taught, included additional international orientated courses and consequently attracted both Dutch and international students. For the US students, the project course was part of a regular major and included no additional international elements. The language of instruction was English. For some students English was their mother tongue; for others it was their second or third language. The project course ran for 10 weeks, and all classes were scheduled online. Students were from different undergraduate programmes and were not familiar with each other. The gender distribution within the US experimental and US control group was comparable, as it was within the NL experimental and control groups.

### Collaborative Learning Assignment and COIL Intervention

The course objectives were assessed by means of a collaborative online learning assignment (i.e., learning and teaching a new motor skill) (see Additional file 1: Appendix A) designed by the NL and US educators. All students from all classes were given this assignment and collaborated in groups to complete it over the course of 10 weeks. Groups were formed randomly by the educators. Each group consisted of two partnering teams that were made up of approximately three students forming a group of six. The collaborative assignment was designed to be highly interdependent, both in the tasks and goals (Johnson & Johnson, 1989) and included team icebreakers tasks as well as five tasks related to course objectives. One task included each team developing an instructional video which they needed to send to their partnering teams. Without the video, partnering teams would not be able to continue with the assignment. Compulsory synchronous online sessions, in which teams and groups carried out their tasks, were scheduled as part of the course work during school time. BlackBoard Collaborate was the chosen online platform used for the online sessions. To encourage active collaboration, students were required to participate actively in the instructional video and in the online sessions and marks were awarded for participation. The fifth part of the assignment included an individual reflection report. Students received a final group assessment score for the complete assignment.

The COIL intervention involved teams of students in the NL and US experimental groups being matched to each other. This resulted in 10 groups (comprised of 5 US teams and 5 NL teams). These groups collaborated on the assignment together and asked each other cultural related questions which is part of the COIL component (see Additional file 1: appendix B). Students within the control groups were matched with students from their own classes to complete the assignment. Within the NL control group this resulted in four groups (comprised of 8 teams) and within the US control group resulted in five groups (comprised of 10 teams).

The researchers took numerous measures to ensure continuity across each of the groups so that all students from all groups had a similar learning environment and experimental setting. This included following the same course and setting the same learning outcomes, an identical assignment (except for the COIL component), all students used the same online platforms (i.e., Blackboard, WhatsApp). In doing so, extraneous variables, that may have created differences in intercultural competence development between the groups, were controlled for. It is also important to note that when signing up for the course, students were not aware whether their class would be in the COIL setup or not. By designing the experiment this way, we tried to isolate the effects of the experimental manipulation (COIL) on the dependent variable (intercultural competence development).

### Measurements and procedure

All students completed an online survey (see Additional file 1: Appendix C) which contained the CQS and the MPQ (see “Introduction”). The survey was administered online during class at the beginning of the project in week 1 (T1). This allowed us to establish a baseline score for each student and check for any differences between the groups prior to the course. The same survey was administered again online at the end of the course, in week 10 (T2), to establish intercultural competence development.

#### Cultural Intelligence Scale

The Cultural Intelligence Scale (CQS) developed by Ang et al. (2007) is a self-report 20-item questionnaire which uses a seven-point Likert scale ranging (1 = strongly disagree; 7 = strongly agree). The subscales of the questionnaire assess the four CQ dimensions:

- *Meta-cognitive* includes four items (e.g., I adjust my cultural knowledge as I interact with people from a culture that is unfamiliar to me, Cronbach’s alpha pre-survey = 0.86, post survey 0.88)
- *Cognitive* includes six items (e.g., “I know the cultural values and religious beliefs of other culture”, Cronbach’s alpha pre-survey = 0.86, post-survey = 0.90)
- *Motivational* includes five items (e.g., “I enjoy interacting with people from different cultures”, Cronbach’s alpha pre-survey = 0.85, post-survey = 0.90)
- *Behavioural* includes five items (e.g., “I vary the rate of my speaking when a cross-cultural situation requires it”, Cronbach’s alpha pre-survey = 0.84, post-survey = 0.86).

#### Multicultural Personality Questionnaire (MPQ)

The MPQ (SF) is a self-report 40-item survey, which uses a five-point Likert scale ranging from 1 “totally not at all applicable” to 5 to “completely applicable”. Each of the five MPQ constructs include eight items (i.e. statements):

- *Cultural Empathy* (e.g., “sympathizes with others;” “Enjoys other people’s stories”, Cronbach’s alpha pre-survey = 0.72, post-survey = 0.78),
- *Flexibility* (e.g., “Wants to know exactly what will happen”, “functions best in a familiar setting”, Cronbach’s alpha pre-survey = 0.77, post-survey = 0.78)
- *Social Initiative* (e.g., “Is often the driving force behind things” “Is inclined to speak out”, Cronbach’s alpha pre-survey = 0.81, post-survey = 0.80),
- *Open-mindedness* (e.g., “has a feeling for what is appropriate in a specific culture”; “likes to imagine solutions for problems”, Cronbach’s alpha pre-survey = 0.76, post-survey = 0.70),
- *Emotional Stability* (e.g., “is insecure”; “is nervous”, Cronbach’s alpha pre-survey = 0.81, post-survey = 0.84)

Two Identifier questions were included in the survey (i.e., student number and lecturer’s name), so that the pre- and post-surveys could be matched. This resulted in a 62-item survey (see Additional file 1: Appendix A). For coherency, a 5-point Likert scale ranging from 1 “not at all applicable” to 5 “completely applicable” was used for the entire survey.

#### Online focus groups

Online focus groups were held at the end of the course to get insight into how students reflected on their learning experience and to examine whether there was evidence of intercultural competence development. One student from each of team took part in a focus group discussion. This resulted in eight focus group sessions i.e., two for each one of the experimental and control groups. Each session consisted of five-six participants. All participants were interviewed online, via Blackboard Collaborate, by three members of the research team. The researcher from the NL interviewed the students from the US and the researchers from the US interviewed the students from the NL. Each session lasted approximately 30–40 min and was recorded with the permission of the participants. The focus group questions were semi-structured and included questions related to the students’ learning experience and collaboration (see Additional file 1: Appendix D). We deliberately asked each group the same set of questions and did not ask direct questions related to intercultural competence as we did not want to prompt responses regarding intercultural competence development. Each recording was transcribed afterwards and used as textual data.

#### Individual reflection reports

At the end of the course, as part of their final assignment, all students were asked to write an individual reflection report. In this report, students were asked to answer a set of questions (see Additional file 1: Appendix E) related to their learning experience, e.g., working online, collaborating in groups, giving feedback and challenges encountered. The reports form all groups were collected and a sample of 56 reports out of 108 (approx. 50% of each group) was used as a data source.

#### Analysis of qualitative data

We took the same deductive approach when analysing both the focus group and reflection report textual data. This involved developing a coding scheme based on the CQS and MPQ constructs developed by Ang et al. (2007) and van der Zee and Van Oudenhoven (2013). To ensure that we took a systematic approach that could be replicated, we used content analysis which is defined as “a research technique for making replicable and valid inferences from texts (or other meaningful matter) to the contexts of their use” (Krippendorff, 2004, p. 18). This process was completed by examining the texts, breaking them down into segments and applying the codes when an example of one of the constructs was identified in each segment. We then quantified the presence of the constructs by counting the number of times an example of each one appeared. We began the coding process using codes based on both the MPQ and CQS. However, due to difficulty applying the MPQ codes, i.e., codes were not transferable, and due to challenges identifying examples of the constructs within the textual data, only the codes based on the CQS were applied to the textual data and not the MPQ (see Table 2). Two external researchers, who were not associated with the study, applied the same coding procedure to 20% of the textual data. The results were compared with our findings which resulted in an 82% inter-coder reliability agreement percentage. We also calculated Fleiss’ Kappa which gave us a 0.80 overall agreement rate. Afterwards, the coders discussed the results and reconciled the differences, and came to a 100% agreement rate. Once the coding scheme was proven to be reliable, we continued coding the remainder of the data. We recorded the number of times an example of each construct appeared in each of the texts and then compared the results to see if there were any differences between the experimental and control groups.

**Table 2:** Coding frame (based on CQS constructs)

Code	Definition	Example
Metacognitive (META)	*the mental capability to acquire and understand cultural knowledge*	Conscious, adjusts cultural knowledge when interacting with others, checks accuracy of cultural knowledge
Cognitive (COG)	*knowledge about cultures and cultural differences*	Knowledge on legal, economic systems, languages rules of other languages, values, religions, marriage systems, arts, non-verbal behaviours
Motivational (MOT)	*the capability to direct and sustain effort toward functioning in intercultural situations*	Enjoy interacting with people from different cultures, confident in socializing with people from different cultures
Behavioural (BEH)	*the capability for behavioural flexibility in intercultural interactions*	Change my behaviour (accent, tone, vocab., speed, nonverbal communication) when a cross-cultural interaction requires it

## Results

### Quantitative data

We statistically compared the survey results of the various groups. 115 students filled in the pre-survey, and 110 students filled in the post-survey. Three students dropped out of the course after the first two weeks. Two students did not complete the post-survey and two students were excluded from the analysis based on answering the middle value ‘moderately applicable’ on respectively 95% and 87% of items, suggesting they did not provide serious answers. This resulted in a final sample of 108 participants who filled in the pre and post survey.

For statistical analysis we transformed the 5-point Likert-scale data to numerical values 1–5 and reversed it for the MPQ questions that were negatively formulated (as recommended by van der Zee et al., 2013). We then checked if the data was complete. For statistical tests we conducted one-tailed tests, with the assumption that the experimental groups would do better than the control groups. The results of our findings are summarized in Table 3.

**Table 3:** Means and standard deviations of Cultural Intelligence Scale (CQS) and Multicultural Personality (MPQ) for pre-survey, post-survey, and gain scores

	US experimental			US control			NL experimental			NL control		
	*N*	*M*	*SD*	*N*	*M*	*SD*	*N*	*M*	*SD*	*N*	*M*	*SD*
CQS presurvey	29	3.19	0.44	31	3.18	0.57	24	3.40	0.58	24	3.38	0.54
CQS postsurvey	29	3.34	0.55	29	3.04	0.69	24	3.52	0.59	24	3.55	0.45
CQS gain score	29	0.18	0.58	29	− 0.10	0.34	24	0.12	0.36	24	0.17	0.35
CQS META gain score	29	0.09	0.80	29	− 0.20	0.54	24	0.05	0.57	24	0.04	0.50
CQS COG gain score	29	0.40	0.87	29	0.09	0.57	24	0.20	0.43	24	0.38	0.56
CQS MOT gain score	29	0.10	0.64	29	− 0.30	0.46	24	0.05	0.51	24	0.08	0.51
CQS BEH gain score	29	0.08	0.92	29	− 0.03	0.64	24	0.15	0.53	24	0.12	0.58
MPQ presurvey	29	3.42	0.40	31	3.35	0.32	24	3.39	0.33	24	3.42	0.32
MPQ postsurvey	29	3.42	0.38	29	3.38	0.27	24	3.38	0.39	24	3.44	0.24
MPQ gain score	29	0.00	0.30	29	0.05	0.18	24	− 0.01	0.24	24	0.02	0.29

CQS: Cultural Intelligence, CQS META: Metacognitive, CQS COG: Cognitive, CQS MOT: Motivational, CQS BEH: Behavioural, MPQ: Multicultural Personality Questionnaire

Because our data was hierarchically nested (i.e., students within teams) and we presumed that the assumption of nonindependence of observations of the dependent variables was violated due to students within teams influencing each other (Cress, 2008; Janssen et al., 2013), we checked the intraclass correlation coefficients (ICCs) of the dependent variables. When ICCs are substantial, traditional tests using analysis of variance may produce biased results and instead multilevel analyses may be better suited for these situations. We found however that all ICCs were low (range = 0.000 and 0.112) and non-significant. We therefore concluded that the assumption of nonindependence was not violated and that the planned *t*-test could be performed.

### Differences in levels of intercultural competence pre intervention

As previously described in the methods section, the students from the NL groups were enrolled in an internationally orientated minor and contained both Dutch and international students, while the US students’ course did not have this international emphasis and all students within the US groups were of US nationality. These elements could lead to the NL students having higher initial intercultural competence. We determined if this was the case before the experiment by testing for significant differences between the groups on the pre-survey.

When we look at the pre-survey scores on the CQS (Table 3), we can see that the scores of the two NL groups are higher than those of the two US groups. An independent samples *t*-test between the two NL groups together and the two US groups together did return a significant result: *t*(106) = − 2.00, *p* = 0.048, *d* = 0.39, indicating that the NL groups indeed had higher initial intercultural competence and were therefore not comparable to the US groups.

When we look at the pre-survey scores of the MPQ (Table 3), the scores show that all groups were comparable on the MPQ at the start of the experiment. The *t*-test between the two NL groups together and the two US groups together was not significant *t*(106) = − 0.27, *p* = 0.786, *d* = 0.05.

Because we found differences in the pre-survey, we decided to analyse the results of the NL and US groups separately. We did this by comparing the increase in CQS and MPQ scores between the US experimental group and the US control group and between the NL experimental group and the NL control group. To do so, we calculated gain scores for all students by subtracting students’ pre-survey scores from their post-survey scores and then comparing the gain scores between the groups.

### Cultural Intelligence Scale (CQS)

Table 3 shows the results of the CQS. An independent samples *t*-test showed that US experimental group students’ gain scores were significantly higher than US control group students’ gain scores (− 0.10), *t*(56) = 2.20, *p* = 0.016 (one-tailed), *d* = 0.58. This result supports our hypothesis that COIL increases intercultural competence.

The NL experimental group increased on the CQS (gain score = 0.12). However, the NL control group showed an even bigger increase (gain score = 0.17). The difference between these two gain scores was not significant *t*(46) = − 0.47, p = 0.641, *d* = 0.14. So, although the NL experimental group increased on the CQS, so did the NL control group and we did not observe the expected effect of a bigger gain score for the NL experimental group than for the NL control group.

We did further investigation on the increase of the groups on the four constructs of the CQS: metacognitive, cognitive, motivational and behavioural. The results are summarized in Table 3 which show the mean gain scores for the experimental and control groups for the four constructs of the CQS. Independent samples t-tests showed that US experimental group students' gain scores were significantly higher than US control group students for the motivational construct: *t*(56) = − 2.75, *p* = 0.004 (one-tailed), *d* = − 0.70. Because we ran multiple tests, we applied the Benjamini–Hochberg procedure, using a false discovery rate of 5%, to avoid statistical errors due to multiple testing (Benjamini & Hochberg, 1995). The significant p-values reported (CQS gain score overall, and CQS motivational gain score), all exceeded the calculated critical values. For the NL students, no differences were observed in the mean gain scores.

### Multicultural Personality Questionnaire (MPQ)

Table 3 shows that on the MPQ, we did not observe any significant differences for the experimental groups than for the control groups. In addition, when we analysed the MPQ constructs, neither of the experimental groups had a bigger increase on any of the constructs compared to the control groups. Considering we had a directional hypothesis and there were no overall differences, we did not do further testing.

### Qualitative Data

Tables 4 and 5 show the breakdown of the coding instances for the deductive coding categories per group for the focus groups and reflection report textual data. The data from the focus groups revealed that higher numbers of CQS coding instances were found for the experimental groups especially in Cognitive CQ. There were 26 instances of Cognitive CQ found for both the US and NL experimental groups and nine Cognitive CQ instances found for the NL control group. There were no CQS coding instances found for the US control group.

**Table 4:** Frequency of CQS constructs identified in Focus Group data

Construct	US Experimental	US Control	NL Experimental	NL Control
Metacognitive	0	0	1	1
Cognitive	26	0	26	9
Motivational	15	0	4	2
Behavioural	10	0	15	1

**Table 5:** Frequency of CQS constructs identified in Reflection Report data

Construct	US experimental	US control	NL experimental	NL control
Metacognitive	1	0	16	3
Cognitive	35	0	13	7
Motivational	24	0	28	4
Behavioural	10	0	1	4

When we analysed the data from the reflection reports, we found comparable results. Higher numbers of CQ instances were found within US and NL experimental groups’ reports, specifically in Cognitive CQ and Motivational CQ. Fewer coding instances of CQ were found in the NL control group and no instances were found within the US control groups’ reflection reports. Examples of these instances can be found in Table 6 and include reports of students comparing cultures, feeling more comfortable with other cultures and showing enthusiasm for learning about other cultures.

**Table 6:** Examples: deductive categories and corresponding excerpts from the Focus Groups (FG) and Reflection Reports (RR) data

Deductive categories	US Experimental	US Control	NL Experimental	NL Control
Metacognitive	*“We kept everything simple, being respectful about their culture and not making assumptions, or asking things that could be offensive”* (FG)	NA*	“*Our group was critical in giving feedback. It was a challenge to give the US students feedback because the Dutch are quite down to earth and direct, so I didn't want to offend them”* (RR)	*“I grew up on 4 different continents, I have experienced a lot of the different cultures, but never analysed them and thought about them in the way we did in this course”* (RR)
Cognitive	*“The similarities in culture were shocking to me. The students from the Netherlands expressed interest in the same artists, shows, and movies as me”* (FG) *“It was fun to hear about life there versus here and how different it is and also how things are handled differently during the COVID”* (FG) *“I was surprised to find out how inexpensive college is in the Netherlands compared to the US.* (RR)		*“We talked about politics. It's very interesting to hear what it's like as an American. The news here puts Trump in a negative spotlight, but in the US, many people vote for Trump, and sometimes you kind of forget how much support he has” (FG))* *“It is weird to hear you can drive when you are 16 in the US, but you cannot drink alcohol when you are 18. And you can die for your country when you are 18, but you cannot have a beer”* (FG)	*“Students in my group was from the U.K and Botswana. That enhanced the experience for me. I could talk to them about what they knew or what they are experiencing in their country. Getting to know what they thought was interesting for me”* (FG) *“During class students who were not Dutch would express their opinions about how things were in their countries. For me the differences were both shocking and enlightening”* (RR)
Motivational	*“I love learning about their culture. That was the coolest part for me”* (FG) *“I have never worked with students from a different country, so this was a first for me. I enjoyed this project because not only did I get to try new things, but I also became friends with my group members” (RR)* *“I’ve always wanted to travel around Europe and meet and interact with people who live in different parts of Europe. Having had this experience makes me more comfortable with the idea, because they seemed a lot like us”* (RR)	NA	*“I was pleasantly surprised by the fact that we got a chance to work with students from USA. it was a great opportunity for both, the Americans and us, to get to know different cultures better” (RR)*	*“I really enjoyed this course. Since I’m Dutch, I live in the Netherlands, and don’t travel much, working with students from different cultural backgrounds was interesting. I learned a lot”* (RR)
Behavioural	*“We were aware of the time difference and made sure we texted at a normal time and were not texting, waking someone up at 3:00am” (FG)* *“English is not their primary language, so we had to make things simpler to understand. That way we weren't running into that barrier. When we did that, everything became easier.”* (FG)	NA	*“You have to ask a couple of questions before you get the real answer with some of the Americans. This took time. Dutchies are very direct, we let you know what the answer is straight away. This was sometimes challenging”* (FG) *“I was ashamed of my English as it’s their native language, but it all went well, and collaboration went really well”* (FG)	*“Because the course was in English, I was pushed outside my comfort zone. This caused me to widen my vocabulary and understanding of the English language”* (RR)

*NA: not available. No CQS coding instances or examples were found for the US control group

### Other observations

When asked to reflect on the course, students from all groups reported that they enjoyed doing the motor learning assignment and communication went well. However, most students from the experimental groups tended to report that collaboration with the US/NL students was the most positive aspect of the course. This was also evident from the high number of Motivational coding instances found in the data within the experimental groups (see examples in Table 6, column 2 & 4). Students from all groups complained about online connection issues and working online as opposed to working face to face. The US students from both groups complained about scheduling issues interfering with trying to meet with each other. Students also reported that they enjoyed working online but would have liked to combine this with meeting in person as they reported it was more difficult to carry out the specific project assignment online and they also missed the face-to-face contact. The NL experimental group also had concerns over their English proficiency i.e., speaking with and understanding the US students.

## Discussion

The purpose of this study was to empirically test the effectiveness of COIL on intercultural competence development in higher education. Our experimental design allowed us to compare developmental changes made by students who had the COIL experience in comparison to students who did not. Overall, the quantitative and qualitative results indicate that COIL increases intercultural competence, in terms of cultural intelligence (CQ). However, the quantitative results showed that the NL control group also increased in CQ. This might be due to the NL students being exposed to other international elements during the course. In the following paragraphs we will further discuss our findings as well as the implications and limitations of this study.

### Findings

When we analysed the Cultural Intelligence Scale (CQS) quantitative data, we saw a significantly bigger increase in intercultural competence for the US experimental group compared to the US control group. The qualitative data (focus groups, reflection reports) further supported this result as we found more evidence of self-reported intercultural competence development within the US experimental group compared to the US control group, in which no evidence was found. The results for the US groups therefore support our hypothesis that intercultural competence is further developed through COIL. Although the NL experimental group did not show a significantly higher gain in intercultural competence compared to the NL control group, the qualitative data showed more coding instances of intercultural competence development in the NL experimental group compared to the NL control group.

There are several possible explanations for these results. First, the NL students were following an international minor, which included the project course in which the experiment was carried out. Given its international focus, this minor might have attracted students who were already interculturally competent and interested in learning about other cultures. This explanation is supported by the fact that the NL students scored higher on the CQS pre-survey compared to the US students. Second, the NL minor also included a culture course which covered topics related to cultural differences. This course could have facilitated further intercultural competence development within the NL groups. Third, given the international orientation of the NL minor, it attracted Dutch and international students. Therefore, both the experimental and control group consisted of a mix of Dutch students and international students. So even in the control group, the students were already potentially collaborating with students from other cultures. This could have increased their intercultural competence even though they were not exposed to the COIL manipulation. On the US side, both the experimental and control groups consisted of only US nationals and their course was not part of an international minor. These factors could explain why we observed a clearer effect of the COIL manipulation with the US groups and explain why we saw no significant differences on intercultural competence development between the two NL groups. However, if this is the case, we would expect that the NL experimental group, who were also influenced by these factors and in addition had the COIL treatment, would have shown an even bigger increase on the CQS compared to the control group, and this was not the case. Furthermore, we do not know how combinations of different internationalisation activities affect intercultural competence development and if these indeed add up or if each one of them is a sufficient condition to reach a certain threshold of intercultural competence above which extra activities have little additional effect. This interaction between different internationalisation activities is an interesting topic for further research.

We did not find any difference between the experimental and control groups on the MPQ. Given that the MPQ measures personality traits which are more stable and change over a longer time, this could explain why no effect was found in our study (i.e., personality doesn’t change in 10 weeks). Although the MPQ has been used to measure intercultural competence development and personality change as a result of an international experience (van der Poel, 2020, Tracey et al., 2016) it might not have been the most suitable tool to use to assess or measure intercultural competence during a COIL course and is perhaps only suitable for measuring development in longitudinal studies (Hofhuis et al., 2020b; Liang & Schartner, 2020) or as a predictor of international aspirations (Van der Zee & Van Oudenhoven, 2001). Given the latter, we checked the results for a correlation between MPQ scores on the pre survey and the increase in CQS. However, this correlation was absent (*r* = 0.04). Other researchers have also found no relationship between the MPQ and intercultural competence and consequently emphasize the need for further research to be carried out using the MPQ as a tool to predict multicultural effectiveness (Martin, 2010).

### Implications

The findings of this research complement the results of earlier studies that investigated the relationship between collaborative learning and intercultural competence development (de Hei et al., 2020; Erez et al., 2013; Liang & Schartner, 2020) and also the results of previous studies on COIL (Naicker et al., 2021; Vahed & Rodriguez, 2020). However, empirical research on COIL remains limited and to our knowledge no studies have been carried out using a quasi-controlled design to measure the effectiveness of COIL. Therefore, by carrying out this study we have started to fill this gap and in doing so aim to lay the groundwork for further empirical research into COIL. The findings of this study also offer practical implications for educators and HEIs, as they provide more insight into which students benefit the most from COIL. For example, implementing COIL within an internationally orientated (English-taught) bachelor’s degree that attracts culturally diverse students might not have the same effect on students’ intercultural development compared to implementing it within a Dutch taught bachelor programme that does not have an international focus, and only attracts Dutch-speaking students. This study also draws attention to the impact of other internationalisation educational practices (e.g., in this case collaboration with international students at the home institution, or a cultural communication course) on intercultural competence development. It would be interesting to explore the effect of COIL in comparison to other internationalisation activities to determine the impact each has on intercultural competence development. We can then determine which practice has the strongest effect or which is more suitable for certain types of students.

In this study we tested the effect COIL has on intercultural competence development, but we did not investigate why and how some students develop intercultural competence more so than others through COIL or what factors might influence intercultural learning. These topics were outside the scope of this study. Future research could involve replicating this study using a larger sample or investigating what factors support meaningful interaction and effective intercultural learning in the instructional design of COIL. Theory and research on Collaborative Learning practices provide us with an interesting foundation to study these elements in the context of COIL. For example, according to Strijbos et al. (2004) the design of the collaborative learning assignment can influence, or hinder interaction. In addition, critical elements within collaborative learning assignments, such as positive interdependence (Johnson & Johnson, 2008), which includes team members sharing common goals, accountability and relying on one another for the outcome, can influence interaction and the effectiveness of cooperation in collaborating teams and consequently learning and should therefore be explored and tested within the COIL practice.

By providing scientific evidence on how students learn through COIL and what factors influence collaboration and intercultural learning, we can provide educators and policy makers with information that can help them make the best decisions when implementing COIL and consequently equip educators with the adequate resources and support when using COIL in their classrooms.

## Conclusion

The study has outlined several important implications for future educational practice and future research. We can conclude that COIL helps students develop intercultural competence, specifically cultural intelligence. However, given the small-scale nature of this study, and the several limitations that have been outlined, further empirical research needs to be carried out on the impact of COIL, and its interaction with other internationalisation practices, to further understand its potential and effectiveness in intercultural competence development.

## Supplementary Information

**Additional file 1.** **Appendix:** Description of project, tasks and cultural questions, survey questions and focus group questions.

## Data Availability

The datasets used and/or analyzed during the current study are available from the corresponding author on reasonable request.
