# Supplementary material for: The effectiveness of Collaborative Online International Learning (COIL) on intercultural competence development in higher education
Source: Int J Educ Technol High Educ. 2023 Jan 24;20(1):5. doi: 10.1186/s41239-022-00373-3 (PMC9870663; doi:10.1186/s41239-022-00373-3)
Supplement: Supplementary file 1 — Additional file 1. Appendix: Description of project, tasks and cultural questions, survey questions and focus group questions. [file 41239_2022_373_MOESM1_ESM.docx]

**Appendix A**

*Collaborative Learning Assignment: Teaching and Learning a Motor Skill*

**Learning Outcomes**

1.Develop the attitude (open, respectful, curious), skills (observing, listening, analysing and reflecting) and knowledge to interact and communicate effectively and show proper behaviour in diverse contexts)

2. Teach a common game/ activity to another team of students

3.Learn a new/develop a new motor skill

4. Examine the changes that occur in teaching and learning as a function of two weeks of practice

5. Reflect on the experiences of both the teacher and the learner in this situation.

6. Demonstrate the ability to adjust or change to best meet the needs of a different situation or environment.

8. Engage in critical reflection on one’s own competencies and development, as well as on the collaboration

**Description**

The project requires students to design an effective instructional video for a specific sport and conduct a small motor teaching and learning experiment. By carrying out this project students experience what it feels like to be both the teacher and the learner. The purpose of this project is to reflect on and examine the changes that occur in people’s learning as they learn and develop a new motor skill.

You will work in groups of six for this project. Your group members will comprise the subjects for the experiment. Each group will be made up of two partnering teams of 3 students. Each team will develop an instructional video for teaching a motor skill to the other team. Groups and teams will be randomly assigned, and you will be informed of which group/team you are in in the first week of the course. Within this project you will take on the role of both the learner and the teacher. To complete the project, you will communicate with each other virtually i.e. through Blackboard Collaborate in class, through WhatsApp etc. You will exchange email addresses with each other and create a WhatsApp group together.

As previously mentioned, each team will develop an instructional video for teaching a motor skill sport. The project is broken up into five tasks. Each week, your team will use class time to work on a part of the project that corresponds with the content covered in class. Your team can also use this time to connect with the rest of your teammates if scheduling allows.

**Tasks***

1. Group Icebreaker and discussion (10 points)
2. Skill selection and Pre project assignment (10 points)
3. Instructional video: Teaching the skill & Giving Feedback (30 points)
4. Practice Video: Learning the skill & Evaluation Group Discussion and Evaluation of Project (35 points) 5.Individual Written Reflection (15 points)

**Total 100 points**

* Full descriptions each of the tasks are found in the course guide.

**Appendix B**

*COIL Cultural Questions*

1. *Driving: typical cars, electric cars, driving laws, and regulations, age of driving? NL versus USA*
2. *Family dynamics: what does a typical family look like? What age do people tend to have children? How many?  NL versus US?*
3. *Marriage: Do many people get married? At what age? What is the ceremony like? Religious/non-religious? Divorce rates? NL versus US?*
4. *Are people religious in the USA/Netherlands? What are the main religions?*
5. *Views on sustainability and climate change:  NL versus USA?*
6. *What are the gun laws in the US versus the Netherlands? How does this affect culture/society?*
7. *Pop culture: what is big now?  in music, style, etc.: NL versus USA?*
8. *Go back to the stereotypical views you wrote about each other at the beginning of the course (assignment 1) – having worked with each other for 7 weeks, do these still apply?*

**Appendix C**

*Online Survey*

1) What is your student number:

2) Name of your lecturer for this course:

**Instructions**

Below you will find various statements. Please take your time to read the statements carefully and answer the statements openly and truthfully.

To what extent do each of the following statements apply to you?

*Choose the answer that is most applicable to you*

1. totally not applicable

2. hardly applicable

3. moderately applicable

4. largely applicable

5. completely applicable

STATEMENTS

3) * Sympathizes with others

| 1. totally not applicable |
| --- |
| 2. hardly applicable |
| 3. moderately applicable |
| 4. largely applicable |
| 5. completely applicable |

4) * Tries out various approaches

| 1. totally not applicable |
| --- |
| 2. hardly applicable |
| 3. moderately applicable |
| 4. largely applicable |
| 5. completely applicable |

5) * Finds it difficult to make contacts

| 1. totally not applicable |
| --- |
| 2. hardly applicable |
| 3. moderately applicable |
| 4. largely applicable |
| 5. completely applicable |

6) * Is reserved

| 1. totally not applicable |
| --- |
| 2. hardly applicable |
| 3. moderately applicable |
| 4. largely applicable |
| 5. completely applicable |

7) * Likes routine

| 1. totally not applicable |
| --- |
| 2. hardly applicable |
| 3. moderately applicable |
| 4.largely applicable |
| 5. completely applicable |

8) * Sets others at ease

| 1. totally not applicable |
| --- |
| 2. hardly applicable |
| 3. moderately applicable |
| 4. largely applicable |
| 5. completely applicable |

9) * Takes the lead

| 1. totally not applicable |
| --- |
| 2. hardly applicable |
| 3. moderately applicable |
| 4. largely applicable |
| 5. completely applicable |

10) * Is often the driving force behind things

| 1. totally not applicable |
| --- |
| 2. hardly applicable |
| 3. moderately applicable |
| 4.largely applicable |
| 5. completely applicable |

11) * Is looking for new ways to attain his/her goal

| 1. totally not applicable |
| --- |
| 2. hardly applicable |
| 3. moderately applicable |
| 4. largely applicable |
| 5. completely applicable |

12) * Makes contacts easily

| 1. totally not applicable |
| --- |
| 2. hardly applicable |
| 3. moderately applicable |
| 4. largely applicable |
| 5. completely applicable |

13) * Keeps calm when things don’t go well

| 1. totally not applicable |
| --- |
| 2. hardly applicable |
| 3. moderately applicable |
| 4. largely applicable |
| 5. completely applicable |

14) * Has a feeling for what is appropriate in a specific culture

| 1. totally not applicable |
| --- |
| 2. hardly applicable |
| 3. moderately applicable |
| 4. largely applicable |
| 5. completely applicable |

15) * Seeks contact with people from a different background

| 1. totally not applicable |
| --- |
| 2. hardly applicable |
| 3. moderately applicable |
| 4. largely applicable |
| 5. completely applicable |

16) * Has fixed habits

| 1. totally not applicable |
| --- |
| 2. hardly applicable |
| 3. moderately applicable |
| 4. largely applicable |
| 5. completely applicable |

17) * Likes to imagine solutions for problems

| 1. totally not applicable |
| --- |
| 2. hardly applicable |
| 3. moderately applicable |
| 4. largely applicable |
| 5. completely applicable |

18) * Is insecure

| 1. totally not applicable |
| --- |
| 2. hardly applicable |
| 3. moderately applicable |
| 4. largely applicable |
| 5. completely applicable |

19) * Wants to know exactly what will happen

| 1. totally not applicable |
| --- |
| 2. hardly applicable |
| 3. moderately applicable |
| 4. largely applicable |
| 5. completely applicable |

20) * Enjoys other people’s stories

| 1. totally not applicable |
| --- |
| 2. hardly applicable |
| 3. moderately applicable |
| 4. largely applicable |
| 5. completely applicable |

21) * Starts a new life easily

| 1. totally not applicable |
| --- |
| 2. hardly applicable |
| 3. moderately applicable |
| 4. largely applicable |
| 5. completely applicable |

22) * Is under pressure

| 1. totally not applicable |
| --- |
| 2. hardly applicable |
| 3. moderately applicable |
| 4. largely applicable |
| 5. completely applicable |

23) * Gets upset easily

| 1. totally not applicable |
| --- |
| 2. hardly applicable |
| 3. moderately applicable |
| 4. largely applicable |
| 5. completely applicable |

24) * Leaves the initiative to others to make contacts

| 1. totally not applicable |
| --- |
| 2. hardly applicable |
| 3. moderately applicable |
| 4. largely applicable |
| 5. completely applicable |

25) * Pays attention to the emotions of others

| 1. totally not applicable |
| --- |
| 2. hardly applicable |
| 3. moderately applicable |
| 4. largely applicable |
| 5. completely applicable |

26) * Looks for regularity in life

| 1. totally not applicable |
| --- |
| 2. hardly applicable |
| 3.moderately applicable |
| 4. largely applicable |
| 5. completely applicable |

27) * Is nervous

| 1. totally not applicable |
| --- |
| 2. hardly applicable |
| 3. moderately applicable |
| 4. largely applicable |
| 5. completely applicable |

28) * Functions best in a familiar setting

| 1. totally not applicable |
| --- |
| 2. hardly applicable |
| 3. moderately applicable |
| 4. largely applicable |
| 5. completely applicable |

29) * Is a good listener

| 1. totally not applicable |
| --- |
| 2. hardly applicable |
| 3. moderately applicable |
| 4.largely applicable |
| 5. completely applicable |

30) * Works according to plan

| 1. totally not applicable |
| --- |
| 2. hardly applicable |
| 3. moderately applicable |
| 4. largely applicable |
| 5. completely applicable |

31) * Is inclined to speak out

| 1. totally not applicable |
| --- |
| 2. hardly applicable |
| 3. moderately applicable |
| 4. largely applicable |
| 5. completely applicable |

32) * Has a broad range of interests

| 1. totally not applicable |
| --- |
| 2. hardly applicable |
| 3. moderately applicable |
| 4. largely applicable |
| 5. completely applicable |

33) * Is apt to feel lonely

| 1. totally not applicable |
| --- |
| 2. hardly applicable |
| 3. moderately applicable |
| 4. largely applicable |
| 5.completely applicable |

34) * Enjoys getting to know others profoundly

| 1. totally not applicable |
| --- |
| 2. hardly applicable |
| 3. moderately applicable |
| 4. largely applicable |
| 5. completely applicable |

35) * Is not easily hurt

| 1. totally not applicable |
| --- |
| 2. hardly applicable |
| 3. moderately applicable |
| 4. largely applicable |
| 5. completely applicable |

36) * Works mostly according to a strict scheme

| 1. totally not applicable |
| --- |
| 2. hardly applicable |
| 3. moderately applicable |
| 4. largely applicable |
| 5. completely applicable |

37) * Notices when someone is in trouble

| 1. totally not applicable |
| --- |
| 2. hardly applicable |
| 3. moderately applicable |
| 4. largely applicable |
| 5. completely applicable |

38) * Senses when others get irritated

| 1. totally not applicable |
| --- |
| 2. hardly applicable |
| 3. moderately applicable |
| 4. largely applicable |
| 5. completely applicable |

39) * Worries

| 1. totally not applicable |
| --- |
| 2. hardly applicable |
| 3. totally |
| 4. largely applicable |
| 5. completely applicable |

40) * Works according to strict rules

| 1. totally not applicable |
| --- |
| 2. hardly applicable |
| 3. moderately applicable |
| 4. largely applicable |
| 5. completely applicable |

41) * I seek people from different backgrounds^[[1]](#footnote-1)^

| 1. totally not applicable |
| --- |
| 2. hardly applicable |
| 3. moderately applicable |
| 4. largely applicable |
| 5. completely applicable |

42) * Is a trendsetter in societal developments

| 1. totally not applicable |
| --- |
| 2. hardly applicable |
| 3. moderately applicable |
| 4. largely applicable |
| 5. completely applicable |

43) * I am conscious of the cultural knowledge I use when interacting with people with different cultural backgrounds.

| 1. totally not applicable |
| --- |
| 2. hardly applicable |
| 3. moderately applicable |
| 4. largely applicable |
| 5. completely applicable |

44) * I adjust my cultural knowledge as I interact with people from a culture that is unfamiliar to me.

| 1. totally not applicable |
| --- |
| 2. hardly applicable |
| 3. moderately applicable |
| 4. largely applicable |
| 5. completely applicable |

45) * I am conscious of the cultural knowledge I apply to cross-cultural interactions.

| 1. totally not applicable |
| --- |
| 2. hardly applicable |
| 3. moderately applicable |
| 4. largely applicable |
| 5. completely applicable |

46) * I check the accuracy of my cultural knowledge as I interact with people from different cultures.

| 1. totally not applicable |
| --- |
| 2. hardly applicable |
| 3. moderately applicable |
| 4. largely applicable |
| 5. completely applicable |

47) * I know the legal and economic systems of other cultures.

| 1. totally not applicable |
| --- |
| 2. hardly applicable |
| 3. moderately applicable |
| 4. largely applicable |
| 5. completely applicable |

48) * I know the rules (e.g., vocabulary, grammar) of other languages

| 1. totally not applicable |
| --- |
| 2. hardly applicable |
| 3. moderately applicable |
| 4. largely applicable |
| 5. completely applicable |

49) * I know the cultural values and religious beliefs of other cultures

| 1. totally not applicable |
| --- |
| 2. hardly applicable |
| 3. moderately applicable |
| 4. largely applicable |
| 5. completely applicable |

50) * I know the marriage systems of other cultures.

| 1. totally not applicable |
| --- |
| 2. hardly applicable |
| 3. moderately applicable |
| 4. largely applicable |
| 5. completely applicable |

51) * I know the arts and crafts of other cultures.

| 1. totally not applicable |
| --- |
| 2. hardly applicable |
| 3. moderately applicable |
| 4. largely applicable |
| 5. completely applicable |

52) * I know the rules for expressing nonverbal behaviors in other cultures

| 1. totally not applicable |
| --- |
| 2. hardly applicable |
| 3. moderately applicable |
| 4. largely applicable |
| 5. completely applicable |

53) * I enjoy interacting with people from different cultures.

| 1. totally not applicable |
| --- |
| 2. hardly applicable |
| 3. moderately applicable |
| 4. largely applicable |
| 5. completely applicable |

54) * I am confident that I can socialize with locals in a culture that is unfamiliar to me.

| 1. totally not applicable |
| --- |
| 2. hardly applicable |
| 3. moderately applicable |
| 4. largely applicable |
| 5. completely applicable |

55) * I am sure I can deal with the stresses of adjusting to a culture that is new to me.

| 1. totally not applicable |
| --- |
| 2. hardly applicable |
| 3. moderately applicable |
| 4. largely applicable |
| 5. completely applicable |

56) * I enjoy living in cultures that are unfamiliar to me.

| 1. totally not applicable |
| --- |
| 2. hardly applicable |
| 3. moderately applicable |
| 4. largely applicable |
| 5. completely applicable |

57) * I am confident that I can get accustomed to the shopping conditions in a different culture.

| 1. totally not applicable |
| --- |
| 2. hardly applicable |
| 3. moderately applicable |
| 4. largely applicable |
| 5. completely applicable |

58) * I change my verbal behavior (e.g., accent, tone) when a cross-cultural interaction requires it.

| 1. totally not applicable |
| --- |
| 2. hardly applicable |
| 3. moderately applicable |
| 4. largely applicable |
| 5. completely applicable |

59) * I use pause and silence differently to suit different cross-cultural situations.

| 1. totally not applicable |
| --- |
| 2. hardly applicable |
| 3. moderately applicable |
| 4. largely applicable |
| 5. completely applicable |

60) * I vary the rate of my speaking when a cross-cultural situation requires it.

| 1. totally not applicable |
| --- |
| 2. hardly applicable |
| 3. moderately applicable |
| 4. largely applicable |
| 5. completely applicable |

61) * I change my nonverbal behavior when a cross-cultural situation requires it.

| 1. totally not applicable |
| --- |
| 2. hardly applicable |
| 3. moderately applicable |
| 4. largely applicable |
| 5.completely applicable |

62) * I alter my facial expressions when a cross-cultural interaction requires it.

| 1. totally not applicable |
| --- |
| 2. hardly applicable |
| 3. moderately applicable |
| 4. largely applicable |
| 5. completely applicable |

**Appendix D**

*Focus Group questions*

- *Describe your experiences with this motor learning project?*
- *Did this experience change the way you think about the course content?*
- *Did you feel that collaborating with a partner team enhanced the project?*
- *Did this project facilitate your ability to work with a diverse group of people?*
- *Did this experience change the way you think about broader issues?*

**Appendix E**

*Individual Reflection Report*

As part of their final assignment, at the end of the course all students were asked to write an individual reflection report. In this report, students were asked to answer a set of questions related to their learning experience.

Examples of Reflection Report Questions:

- *Did you enjoy this course?*
- *What did you learn from this course?*
- *How did you experience working in a team and teaching and giving feedback to a partnering team? Challenges, advantages, Group dynamics and differences?*
- *How did you experience receiving positive and critical feedback on your video from your teammates? Were there differences in how each group gave feedback?*
- *how did you find the experience in general of working with students online?*
- *If you could do this assignment again. What would you do differently?*

1. This question was a duplication and replaced the original MPQ question Takes initiatives [↑](#footnote-ref-1)
